# Validation of Electromagnetic Field Sensor Performance Through Porcine Skulls: Implications for Neurostimulation and Recording Techniques

**DOI:** 10.7759/cureus.82032

**Published:** 2025-04-10

**Authors:** Imran Siddiqi, Raphia Rahman, James Brazdzionis, Paras Savla, Jordan M Hough, Devendra K Agrawal, David Connett, Dan E Miulli

**Affiliations:** 1 Neurosurgery, Riverside University Health System Medical Center, Moreno Valley, USA; 2 Internal Medicine-Pediatrics, Loma Linda University Medical Center, Loma Linda, USA; 3 Translational Research, Western University of Health Sciences , College of Osteopathic Medicine of the Pacific, Pomona, USA; 4 Family Medicine, College of Osteopathic Medicine, Des Moines University, West Des Moines, USA

**Keywords:** emf, emf brain injury, moderate tbi, pemf, tbi models

## Abstract

Recent technological advancements have led to the development of portable helmet systems equipped with induction sensor stimulators for non-invasively monitoring neural electromagnetic fields in real-time. The helmet incorporates a Mu-metal shield, a material designed to block low-frequency electromagnetic fields and to reduce external interference. This study utilized an adult pig model to validate the ability of these sensors to record and stimulate neural activity through pig skulls, which closely mimic human cranial anatomy. Sensor-stimulators, which both detect and deliver electromagnetic stimulation, were integrated into the helmet for neural activity monitoring. Employing proprietary BS-1000 induction sensor stimulators integrated into a custom-designed helmet, our research focused on the efficacy of transmitting and modulating electromagnetic fields (EMFs) beyond the varied thicknesses of the pig skull. Induction sensors, a type of electromagnetic field sensor, were used to measure neural signals non-invasively.

The experimental setup included measuring EMF responses at baseline and under conditions of incremental cranial barrier thicknesses, assessing both the recording and stimulating capabilities of the system. Results indicated that the EMF penetrated the swine skull and that the sensors maintained signal integrity and functionality despite increases in bone thickness of the pig skull compared to humans, successfully capturing and stimulating neural activity across all tested scenarios. These findings demonstrate the potential of this technology for non-invasive neuromodulation and neural monitoring. Its application in traumatic brain injury (TBI) research could facilitate real-time assessment of neural function and aid in the development of targeted therapeutic interventions.

## Introduction

Recent technological advancements have enabled the non-invasive, real-time monitoring of neural electromagnetic fields (EMFs) in human subjects through the use of a novel, portable helmet system. Current EMF-based technologies for neural monitoring and stimulation often require highly controlled environments, such as magnetically shielded rooms, to reduce external interference. Traditional EMF-based neural monitoring systems, such as magnetoencephalography (MEG), rely on superconducting quantum interference devices (SQUIDs) housed within magnetically shielded rooms to minimize external electromagnetic interference. These setups use layers of Mu-metal shielding and passive cancellation techniques to isolate neural signals from background noise. Additionally, existing systems struggle with signal attenuation when applied through biological barriers like the skull, limiting their clinical and experimental applicability. This study addresses these limitations by demonstrating the ability of a portable, helmet-mounted EMF sensor-stimulator system to effectively detect and stimulate neural activity through varied skull thicknesses without the need for extensive shielding.

This helmet is equipped with induction sensors capable of distinguishing various neural activities, ranging from emotional states to motor behaviors, from baseline activity, without the encumbrance of traditional MEG setups that require shielded rooms [1-5]. However, the efficacy and versatility of these technologies, particularly in pathological states or through different mediums such as animal skulls, remain underexplored.

To address this gap, this study investigates the application of advanced EMF recording and stimulation technology using an adult pig, a well-established proxy for human neurophysiological studies due to its similar cranial anatomy and brain function, especially in the context of traumatic brain injury (TBI) research [6,7]. Specifically, our research aims to validate whether these portable EMF sensor-stimulators can effectively record and stimulate neural activity through the dense structure of a pig skull.

The helmet used in this study is specifically engineered to optimize EMF signal fidelity while minimizing noise interference. It features a dual-layered Mu-metal shield, interlaced copper mesh, and strategic air gaps to isolate the sensors from external EMF interference. This design ensures that the sensor-stimulator can operate effectively in various clinical and non-clinical environments without the need for traditional, bulky shielded setups [8,9].

Unlike previous studies that primarily focused on EMF signal propagation through soft tissues or uniform skull models, this study systematically examines signal transmission across varying skull thicknesses. By using multiple layers of biological bone, it provides a more comprehensive assessment of EMF behavior in realistic cranial conditions, enhancing the translational potential of this technology for both research and clinical applications. This paper details our methodology for evaluating the transmission and modulation of EMFs through pig skulls, simulating real-world conditions where direct brain access is impractical. We hypothesize that skull thickness will not significantly attenuate EMF signal penetration, allowing for consistent neural recording and stimulation across varying cranial densities. This will be assessed by comparing signal amplitude, frequency integrity, and stimulation efficacy across different skull thickness conditions.

## Materials and methods

In this study, we employed BS-1000 proprietary induction sensors integrated with EMF signal generators and stimulators provided by Quasar Federal Systems (San Diego, CA). These components were incorporated into a custom-designed helmet with EMF channels, following methodologies previously outlined [1-5,8-9]. The helmet’s construction featured dual-layered Mu-metal (MuMETAL®, Magnetic Shield Corporation, Bensenville, IL), a 2.5 cm air gap, and layers of interlaced copper mesh, all encased within plastic shells. Sensors were strategically embedded within the 15 Mu-metal-shielded EMF channels of the helmet.

Data acquisition was managed using a 16-bit data card from National Instruments (Austin, TX), operating at a 5 kS/s (kilo-samples per second) rate. The signal processing setup included a 2 kHz low-pass filter and a 10x gain module. The sensors were fine-tuned to a sensitivity of 1 pT/√Hz at 1 Hz, enabling the capture of a broad range of EMF signals from 1 Hz to 2 kHz. Waveform analysis was conducted using Igor® Pro version 8 (WaveMetrics, Lake Oswego, OR), employing a fast Fourier transform (FFT) algorithm to convert time-domain signals into frequency-domain signals. The analysis focused on variations in waveform morphology, particularly peaks, valleys, and slopes, to detect potential interference caused by skull barriers. To validate signal integrity and consistency, FFT analysis and baseline comparison with known EMF sources were employed to confirm accurate detection and minimal interference.

Ethical approval for the human control experiment was obtained from the Institutional Review Board of Arrowhead Regional Medical Center (approval no. 22-51), and all participants provided informed consent before participation. The applied EMF stimulation parameters were chosen to remain within established safety limits set by the International Commission on Non-Ionizing Radiation Protection (ICNIRP) to minimize any potential side effects. The experimental procedure began with a baseline EMF data recording from a control, which consisted of recordings from a healthy male human subject at rest. These were obtained for 30 seconds, with the helmet suspended directly above the subject’s head without making contact. This was followed by a 30-second stimulation at frequencies of 2.5 Hz and 5.5 Hz, with a 500 mV positive offset and a 1-volt amplitude, delivered through sensor 818 positioned over the left motor cortex to simulate neural activation. Post-stimulation, EMF signals were recorded for an additional 30 seconds to evaluate the immediate effects of the stimulation.

A fresh adult pig skull was obtained from a local abattoir and immediately processed for use in the experiment to preserve its natural conductivity properties. The Western University of Health Sciences Institutional Animal Care and Use Committee issued approval (protocol no. R23IACUC003). The pig was selected within a standardized age range of six to eight months to ensure cranial maturity and minimize variability in bone density. All skull samples were kept hydrated during the experiment to prevent drying effects that could alter EMF signal transmission. A single pig skull was used, with excised bone discs stacked to simulate varying skull thicknesses. This approach was chosen to better reflect real-world applications, where individual differences in skull thickness may impact EMF signal transmission. Environmental factors, including temperature (maintained at 22°C) and humidity (kept at 50%), were controlled within standard laboratory conditions to minimize external influences on signal recordings. Prior to each experimental session, sensors were calibrated using a standardized EMF reference signal to ensure consistent sensitivity and accuracy across trials

To simulate varying thicknesses of a pig skull, adult pig skulls were segmented to create plugs of different thicknesses (2/8 inch, 3/8 inch, 6/8 inch, and another 6/8 inch), resulting in a composite total thickness of 2⅛ inches. These plugs were sequentially arranged within the EMF channels to progressively increase the barrier’s density. Each plug fully occupied the channel without leaving any gaps. The EMF recordings and stimulation exercises were conducted through these skull setups, with the subject tapping at a rhythmic frequency of 2 Hz. Special attention was given to sensor 818, which was used both for recording and delivering stimulation at 2.5 Hz and 5.5 Hz, with a 1000 mV amplitude over a duration of three minutes.

Signal integrity and any attenuation caused by the skull barriers were meticulously assessed. Both quantitative and qualitative analyses focused on changes in peak, valley, frequency, and amplitude of the recordings across the varying skull thicknesses. Data analysis focused on identifying consistent trends in signal behavior. Variations were examined qualitatively and quantitatively by assessing percentage changes in amplitude relative to baseline measurements. A change was considered notable if amplitude variations exceeded 10% across conditions, as this threshold indicated a meaningful deviation in signal integrity. Data visualization was performed using FFT spectral analysis, and trends in amplitude reduction were examined qualitatively rather than through formal statistical testing. This comprehensive analysis was designed to determine any significant interference or attenuation of the EMF signals due to the skull, thereby providing empirical data on the efficacy of the helmet and sensor system in operating through biological barriers. 

## Results

In our study, we employed proprietary induction sensor stimulators (BS-1000) provided by Quasar Federal Systems to investigate their ability to record and stimulate neural EMFs through varying thicknesses of pig skull, a model that approximates the human cranial structure. The primary focus of this research was using pig skull plugs to simulate increased cranial thickness, ranging incrementally from 2/8 inch to a final thickness of 2⅛ inches. Attenuation was quantified by comparing the signal amplitude and frequency stability across different skull thicknesses using normalized attenuation ratios.

**Figure 1:**
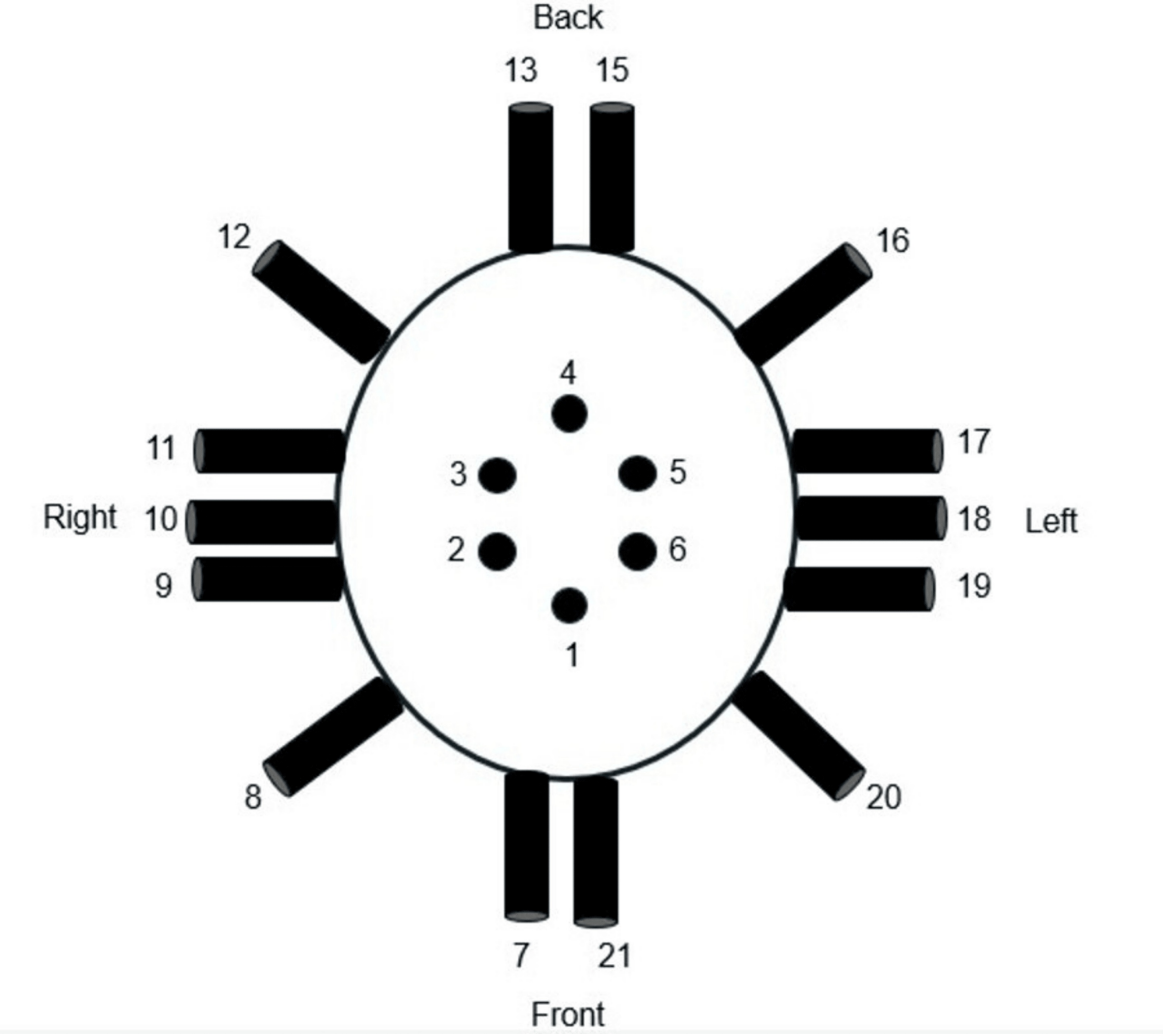
Sensor configuration of the helmet

**Figure 2:**
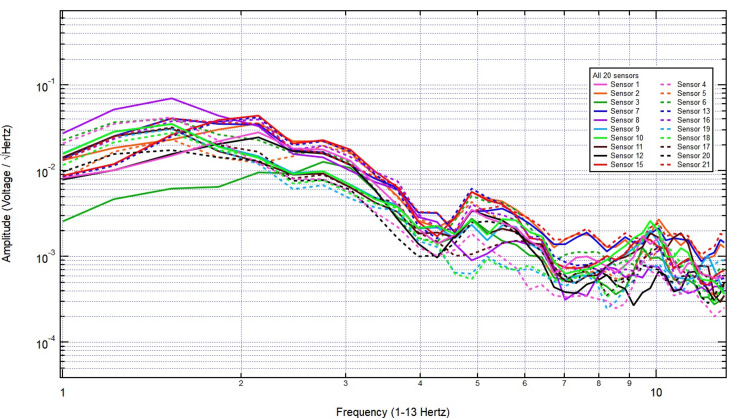
Baseline EMF recording of healthy test subject EMF: Electromagnetic field

The EMF recordings obtained from helmet-mounted sensors were analyzed against a baseline recording from a healthy male volunteer at rest. Figure 1 shows a schematic diagram of the helmet sensor topography. The lightweight helmet is equipped with 20 EMF sensors. Each sensor is wrapped in Mu-metal foil and positioned 9 cm above the scalp, with the positive side oriented toward the scalp. The baseline recording of the human subject displayed distinct frequency-dependent variations with notable peaks, indicating the presence of biologically generated EMF signals (Figure 2).

**Figure 3:**
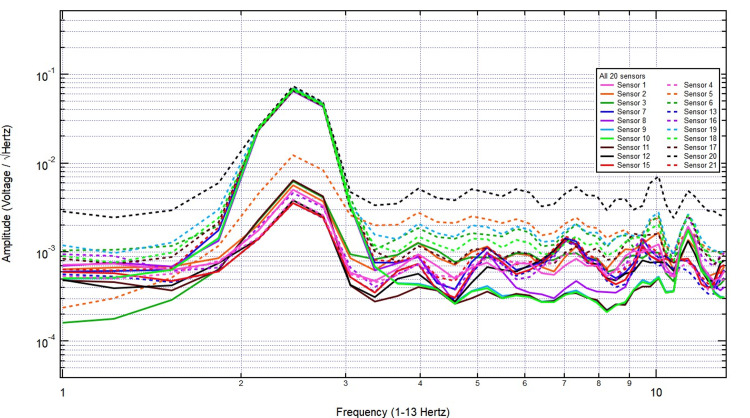
The EMF measurements with 2.5 hertz stimulation without bone interference EMF: Electromagnetic field

The increased amplitude at specific frequencies suggests physiological sources, potentially linked to neural or other biological activity. These findings highlight clear differences between the control and human recordings, reinforcing the sensitivity of the helmet-mounted sensors in detecting biologically relevant electromagnetic patterns. The EMF recordings were analyzed under stimulation conditions with increasing thicknesses of pig skull plugs to assess signal attenuation. The baseline measurement in Figure 3, taken with no bone, exhibited clear frequency peaks with consistent amplitude, representing the unattenuated signal.

**Figure 4:**
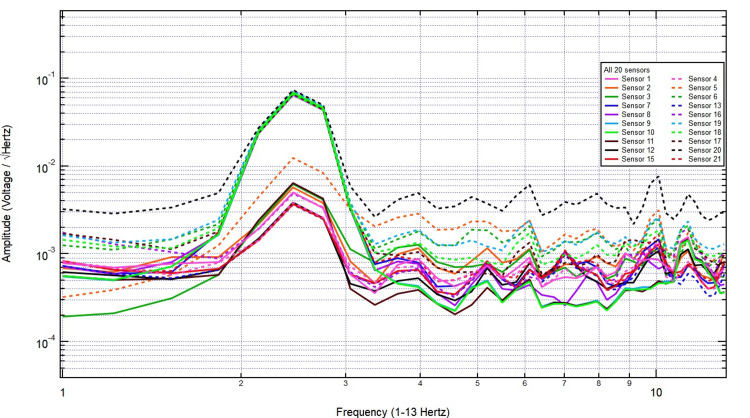
The EMF measurements with 2.5 herz stimulation through 2/8 inch thick bone EMF: Electromagnetic field

**Figure 5:**
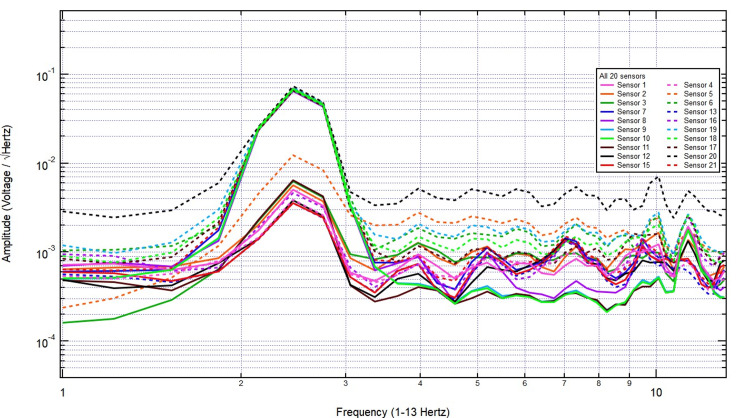
The EMF measurements with 2.5 herz stimulation through 5/8 inch thick bone EMF: Electromagnetic field

Introducing a 2/8-inch bone layer did not cause significant attenuation, as the signal maintained its amplitude across key frequency bands, with only minor variations likely due to natural fluctuations or reflections at the bone interface (Figure 4). As the thickness increased to 2/8 + 3/8 inches, the overall signal amplitude remained largely unchanged, and the frequency distribution retained its structure, indicating minimal absorption or scattering effects from the additional bone (Figure 5).

**Figure 6:**
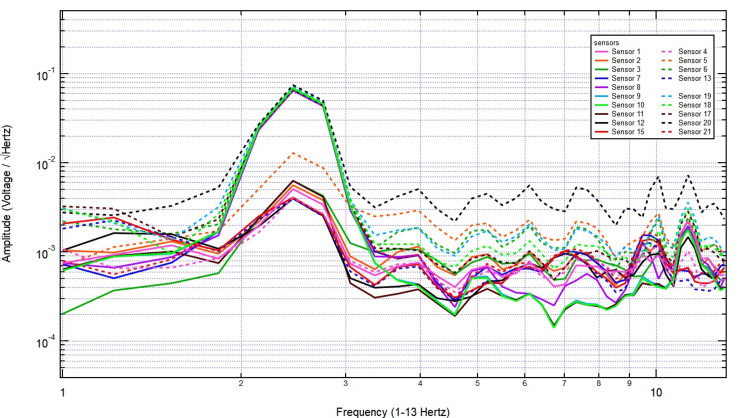
The EMF recordings with 2.5 hertz stimulation through 1 3/8 inch skull barrier EMF: Electromagnetic field

**Figure 7:**
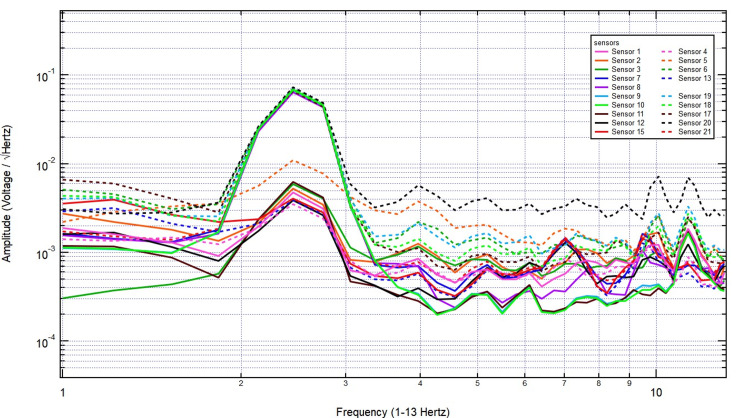
The EMF recordings with 2.5 hertz stimulation through 2 1/8 inch thick skull barrier EMF: Electromagnetic field

Even with further increases to 1 3/8 inches, as shown in Figure 6, the recorded signal continued to display comparable amplitude and frequency characteristics, reinforcing that EMF waves are not significantly attenuated by the skull. Finally, with the thickest skull plug tested (2 1/8 inches), the signal remained stable, with no clear trend of decreasing amplitude as bone thickness increased (Figure 7). These findings confirm that the skull does not act as a major barrier to EMF wave transmission, supporting the feasibility of utilizing the EMF sensors employed with a skull barrier.

**Figure 8:**
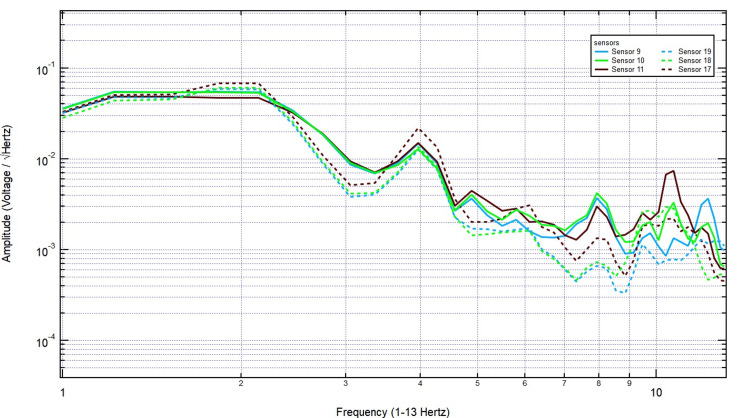
The EMF recordings during right hand tapping without additional skull barrier EMF: Electromagnetic field

The EMF recordings were then analyzed under right-hand tapping conditions, with sensors 17-19 positioned over the left hemisphere (sensor 18 specifically over the left motor cortex) and sensors 9-11 over the right hemisphere. The baseline measurement taken with no bone displayed distinct EMF activity, with strong signal amplitude over the left motor cortex, as expected due to its involvement in right-hand movement (Figure 8).

**Figure 9:**
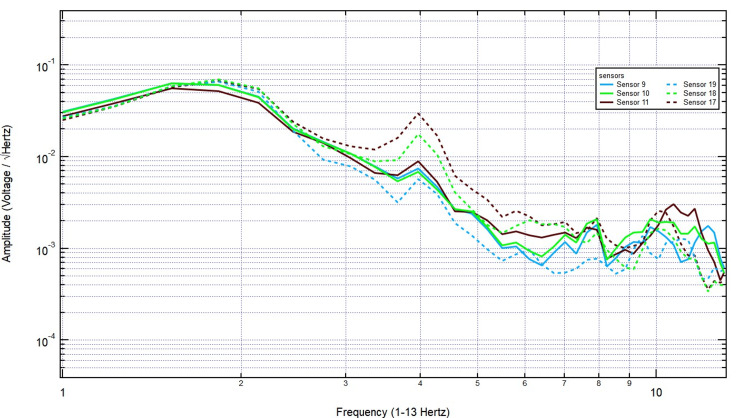
The EMF recordings with right hand tapping and 2/8 inch skull barrier Sensors 17-19 were positioned over the left hemisphere (sensor 18 over the left motor cortex) and sensors 9-11 over the right hemisphere. Introducing the bone layer did not significantly change the recorded signals, as the amplitude and frequency patterns remained stable, especially over sensor 18, suggesting minimal attenuation. EMF: Electromagnetic field

**Figure 10:**
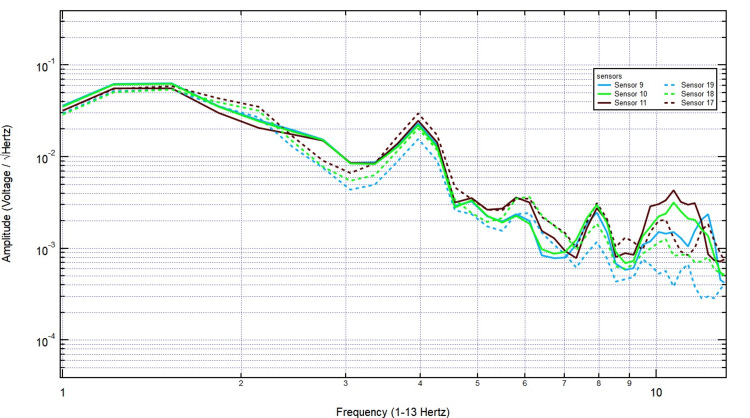
The EMF recordings with right hand tapping with 5/8 inch skull barrier Sensors 17-19 were positioned over the left hemisphere (sensor 18 over the left motor cortex) and sensors 9-11 over the right hemisphere. As the skull thickness increased to 2/8 + 3/8 inches, the signal maintained a stable amplitude with no significant disruption in frequency distribution, further supporting the idea that EMF signals generated by motor activity effectively penetrate bone. EMF: Electromagnetic field

Introducing a 2/8-inch bone layer did not significantly alter the recorded signals, as the amplitude and frequency patterns remained consistent, particularly over sensor 18, indicating minimal attenuation (Figure 9). As skull thickness increased to 2/8 + 3/8 inches, the signal continued to show stable amplitude, with no major disruption in frequency distribution, reinforcing the idea that EMF signals generated by motor activity penetrate bone effectively (Figure 10).

**Figure 11:**
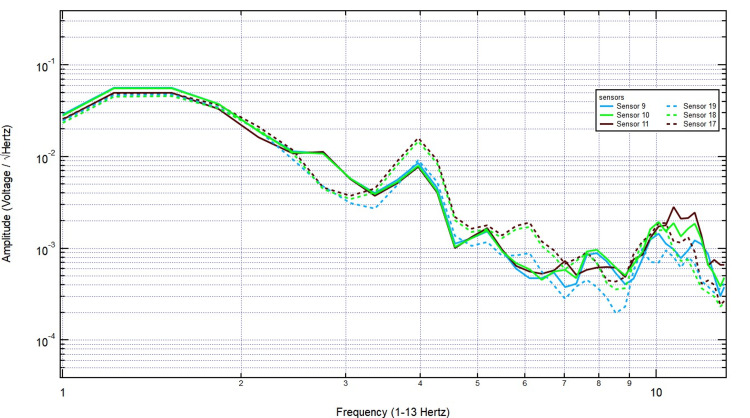
The EMF recordings with right hand tapping and 1 3/8 inch skull barrier Sensors 17-19 were positioned over the left hemisphere (sensor 18 over the left motor cortex) and sensors 9-11 over the right hemisphere. Even with an increase to 1 3/8 inches of skull thickness, key frequency components remained intact, and no systematic decline in amplitude was observed. EMF: Electromagnetic field

**Figure 12:**
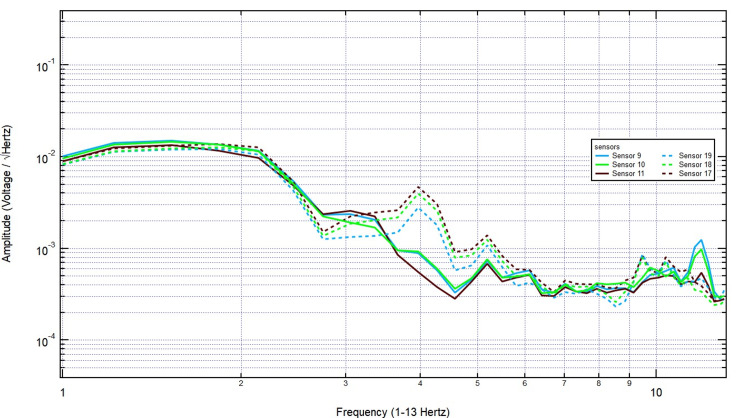
Electromagnetic field recordings with right hand tapping and 2 1/8 inch additional skull barrier Sensors 17-19 were positioned over the left hemisphere (sensor 18 over the left motor cortex) and sensors 9-11 over the right hemisphere. The EMF signal detection was with maximum skull thickness. With the thickest skull plug (2 1/8 inches), signals remained clearly detectable across both hemispheres, particularly over the left motor cortex, indicating that increased bone thickness did not significantly attenuate EMF activity associated with motor function. EMF: Electromagnetic field

As shown in Figure 11, even with further increases to 1 3/8 inches, the key frequency components remained present, and no systematic decline in amplitude was observed. Finally, with the thickest skull plug (2 1/8 inches), signals remained clearly detectable across both hemispheres, particularly over the left motor cortex, demonstrating that bone thickness did not significantly dampen EMF activity associated with motor function (Figure 12). These findings support the conclusion that EMF signals related to right-hand tapping propagate effectively through the skull, further validating the potential of these sensors for further studies utilizing human and porcine subjects.

## Discussion

Our study aimed to validate the effectiveness of BS-1000 induction sensor stimulators, integrated within a custom-designed helmet, for accurately recording and stimulating neural EMFs through varying thicknesses of pig skull, a model for human cranial anatomy [6]. Previous studies have investigated similar EMF-based neurostimulation technologies in healthy human subjects, demonstrating their feasibility for non-invasive neural monitoring. However, the present study extends this by specifically examining signal penetration through bone. The results confirm that the sensors can overcome significant biological barriers, maintaining EMF signal detection and stimulation capabilities even as skull thickness increased from 2/8 inch to 2⅛ inches. The consistent detection of EMF peaks in the 2 Hz to 3 Hz range, corresponding to the tapping stimulus frequencies, highlights the robustness of this sensor technology in penetrating cranial barriers. These findings provide critical validation for applying this sensor system in advanced neurophysiological studies with future pig models and clinical settings.

Pigs, with their cranial anatomy and brain function closely resembling those of humans, serve as invaluable proxies for studying human neurological conditions [7]. The present study demonstrates that the ability to non-invasively record and stimulate neural activity through the skull offers significant methodological advantages. This is particularly relevant for research into TBI, neurodegenerative diseases, and other neurological disorders requiring real-time, in vivo analysis. The successful performance of these sensors in penetrating pig skulls lays the foundation for future studies involving varied neural stimuli and pathological conditions to fully explore the technology's potential.

One of the primary concerns in electromagnetic neural monitoring is the potential attenuation of signal amplitude as it passes through biological tissue, particularly the cranial bone. Our findings demonstrate that EMF signals remain robust across a range of skull thicknesses, from 2/8 inch to 2 1/8 inches. The comparison between the baseline (no bone) and progressively thicker skull samples revealed only minor variations in amplitude, with no clear trend of signal degradation. Even at maximum tested skull thickness, biologically generated EMF signals were reliably detected, supporting the conclusion that the skull does not significantly obstruct electromagnetic wave transmission.

During right-hand tapping trials, the sensors positioned over the left hemisphere, particularly sensor 18 over the left motor cortex, consistently recorded strong EMF activity. The introduction of incremental skull thickness did not alter the fundamental frequency distribution or amplitude, indicating that skull interference in these recordings is minimal. The consistency of these results across varying skull thicknesses reinforces the hypothesis that EMF signals associated with motor activity propagate effectively through bone, making this technique suitable for non-invasive monitoring of cortical activity.

Another key aspect of our study was evaluating the ability of the induction sensors to stimulate neural activity through increasing skull thickness. The results demonstrated that stimulation effects were consistently observed across all tested conditions, with no clear attenuation of efficacy. This suggests that the applied stimulation can reliably penetrate the skull and interact with underlying neural circuits. The ability to both detect and stimulate neural activity across cranial barriers is particularly relevant for applications in neurosurgery, where precise, non-invasive modulation of brain activity is desirable.

Variability in measurements was observed across repeated trials, primarily due to minor fluctuations in sensor positioning and external electromagnetic interference. However, trends in amplitude and frequency remained consistent across conditions, reinforcing the robustness of the findings. No statistical significance testing or confidence interval calculations were performed, as the study focused on identifying qualitative and percentage-based variations in signal attenuation. Potential sources of measurement artifacts, including sensor misalignment, skull disc positioning inconsistencies, and environmental EMF noise, were minimized through careful placement of the helmet and repeated signal acquisition trials to ensure reproducibility.

Neurological injuries such as TBI present complex challenges, particularly due to the difficulty of assessing cortical and subcortical neural circuits in real-time. Traumatic brain injury is a prevalent condition that contributes to nearly one-third of trauma-related deaths in the United States. The high morbidity, mortality, and associated costs underscore the urgent need for effective treatments [10]. However, understanding TBI pathophysiology is hindered by the brain's intricate healing mechanisms and the lack of large, translationally relevant animal models.

To address these challenges, we previously developed a controlled cortical impact (CCI) swine model of TBI [8,9, 11]. This model incorporates a magnetically shielded helmet with EMF channels and induction sensor stimulators, enabling non-invasive monitoring of brain activity both before and after injury. The injured cortical tissue disrupts neuronal signaling, consistent with TBI pathophysiology. Pre- and post-operative measurements demonstrate altered activity patterns, validating the model’s translational relevance.

Importantly, our previous findings also suggest the potential of applying EMF stimulation technologies to target abnormalities in neuronal circuits caused by TBI. Post-injury baseline EMF patterns appeared altered, and AI modeling indicated that post-stimulation neuronal activity began to realign with preoperative baseline patterns. These results suggest that real-time, non-invasive EMF measurements could guide targeted stimulation to correct signal abnormalities, offering a novel approach for treating cortical injuries [8,9,11].

Research involving Yucatan minipigs has demonstrated the therapeutic potential of targeted neural stimulation in promoting recovery after TBI [11]. These findings emphasize the importance of further exploration into EMF modulation as a valuable therapeutic strategy for restoring disrupted neural circuits caused by injury, stroke, or other neurological disorders. In our previous studies, we successfully demonstrated the feasibility of EMF-based neural monitoring and stimulation using a live pig model, confirming that EMF signals can be reliably recorded and modulated in vivo. However, these studies did not systematically evaluate the effects of skull thickness on signal transmission [8,9,11].

The present study builds upon this prior work by introducing a controlled, variable-thickness skull model to assess whether increasing bone density impacts EMF penetration. Our findings indicate that signal transmission remains consistent across different skull thicknesses, reinforcing the robustness of this technology for non-invasive neural monitoring. This is the first study to explicitly demonstrate that skull thickness does not significantly attenuate EMF signals, supporting the applicability of this method across individuals with varying cranial structures.

Our study highlights the promising role of the sensor-stimulator helmet system in detecting and stimulating neural activity through an intact skull, even under conditions of exaggerated skull thickness. This non-invasive tool holds significant promise for advancing TBI research and clinical applications. Expanding research in this area is critical to improving patient outcomes and developing new therapeutic strategies for neurological injuries.

Although our results were broadly consistent with our expectations, minor fluctuations in signal amplitude were observed across repeated trials. These variations were likely due to slight differences in skull disc positioning and minor external electromagnetic interference rather than intrinsic attenuation by the bone itself. Repeated recordings ensured that these effects did not significantly alter the overall findings. Future work will incorporate real-time compensation techniques to further minimize external interference and improve signal consistency in more dynamic biological environments.

Limitations

While this study demonstrates the feasibility of EMF-based neural monitoring through biological bone barriers, several limitations must be considered. First, only a single pig skull was used, with stacked bone discs to simulate different thicknesses. Although this approach allowed for controlled comparisons, it does not fully replicate the structural complexity of an intact skull with sutures and curvature. Additionally, the study did not assess variability across multiple biological specimens, which may influence the generalizability of the results to human subjects. Future studies incorporating live animal models will be essential to confirm the translational applicability of these findings.

Second, while our study demonstrated that skull thickness does not significantly attenuate EMF signals, potential variability in individual skull composition, such as differences in density, porosity, and mineral content, could impact signal transmission in a clinical setting. Further research is needed to assess the influence of these factors across a diverse human population.

Third, the potential impact of soft tissues, cerebrospinal fluid, and blood flow was not included in this model. These factors may affect EMF transmission in live subjects, necessitating future studies in intact animal models or human participants. Furthermore, while environmental variables such as temperature and humidity were controlled, external electromagnetic interference could still contribute to minor fluctuations in signal recordings.

Fourth, the study did not employ statistical significance testing, relying instead on qualitative and percentage-based analyses. Future work should incorporate formal statistical assessments to better quantify variations in EMF signal strength and attenuation under different conditions. Addressing these limitations will be essential for refining the technology and validating its applicability in clinical and real-world settings.

Fifth, there was potential for sensor placement inconsistencies in this study. While care was taken to ensure uniform positioning of the helmet-mounted sensors, minor variations in alignment across trials could have contributed to slight fluctuations in recorded signals. Future studies should explore more precise sensor mounting techniques or automated alignment systems to improve consistency. Additionally, real-world applications may introduce additional noise sources, including movement artifacts, environmental electromagnetic interference, and variations in sensor placement.

Finally, while the study supports the feasibility of non-invasive EMF-based neuromodulation, the long-term effects of electromagnetic stimulation on neural tissue remain to be fully understood. Further investigations should focus on safety, potential neural adaptation, and optimal stimulation parameters to ensure clinical efficacy and patient safety. Addressing these limitations through continued research will be critical in refining the application of helmet-mounted EMF sensors for neuromonitoring and neuromodulation in both experimental and clinical contexts.

## Conclusions

This study provides compelling evidence that the BS-1000 induction sensor stimulators can effectively detect and stimulate neural EMFs through varying skull thicknesses. The findings confirm the robustness of this technology in overcoming biological barriers and its potential for non-invasive applications in neurosurgery, TBI research, and neurophysiological monitoring. The successful validation in pig models establishes a foundation for future translational research, with implications for both clinical and therapeutic advancements. These findings lay the groundwork for the clinical translation of EMF-based neuromonitoring and neuromodulation, particularly in non-invasive TBI assessment and therapeutic interventions. Further investigations are necessary to refine the technology, address existing limitations, and explore broader applications in neurological assessment and treatment. Future research will build upon our previous studies using live pig models to further evaluate EMF-based neural monitoring and stimulation in in vivo conditions. Additionally, planned clinical trials in human participants will assess the technology’s efficacy, safety, and translational potential for non-invasive neuromodulation and neurological diagnostics. These investigations will be critical for refining the system’s applicability in clinical and research settings.
